# Evolution of the general practice receptionist role and online services: a qualitative study

**DOI:** 10.3399/BJGP.2024.0677

**Published:** 2025-08-27

**Authors:** Stephanie Stockwell, Helen Atherton, Carol Bryce, John Campbell, Christine Marriott, Emma Pitchforth, Bethan Treadgold, Rachel Winder, Jennifer Newbould

**Affiliations:** 1 RAND Europe, Cambridge, UK; 2 Unit of Academic Primary Care, Warwick Medical School, University of Warwick, Coventry, UK; 3 Primary Care Research Centre, Aldermoor Health Centre, University of Southampton, Southampton, UK; 4 Exeter Collaboration for Academic Primary Care, University of Exeter Medical School, Faculty of Health and Life Sciences, Exeter, UK; 5 Patient and Public Contributor, National Institute for Health and Care Research Applied Research Collaboration, South West Peninsula Patient Engagement Group, UK

**Keywords:** general practice, medical receptionists, digital technology, focused ethnography, qualitative research

## Abstract

**Background:**

General practice receptionists are perceived as the ‘gatekeepers’ to primary care services and are central to managing patient demand and facilitating patient care. This role is evolving and becoming increasingly complex in a digital world.

**Aim:**

To consider the growing role of patient-facing online services and the impact these services have on the role of the general practice receptionist.

**Design and setting:**

A focused ethnographic case study was undertaken in eight general practices across England and 19 stakeholder interviews took place.

**Method:**

Focused ethnographic case study and stakeholder interviews were conducted between September 2021 and July 2022.

**Results:**

The receptionist role looks different across practices, but is now more varied and less repetitive than it has been historically. The volume of patients and number of channels by which patients contact the practice means that receptionists are dealing with increasingly complex demand management and navigation to appropriate services. This now includes online services, which has created a new element to the receptionist role — digital facilitation. The role is also largely navigated by the receptionists without any formal training and staff are mostly expected to learn on the job from other receptionists, leading to inconsistent practices.

**Conclusion:**

The digitalisation of healthcare services impacts the workflow and consistency in task completion of general practice receptionist staff and has potential implications regarding job satisfaction and retention. In addition, the knowledge and skills required to fulfil this role are evolving and therefore may have recruitment and training implications.

## How this fits in

The introduction of online systems and services into general practice and the impact on general practice staff has been considered from a clinician perspective, but comparatively little is known about how these introductions have affected the receptionist role. This study highlights that the use of online services is leading to an evolution of the general practice receptionist role. The role is becoming increasingly complex as practices use multiple online systems, which impacts demand management and navigation aspects of the role. Online systems have variable consequences on workload for receptionists, which has potential implications for workflow, consistency of task completion, job satisfaction, and retention and recruitment of these key staff members.

## Introduction

The general practice receptionist is one of the most ‘visible’ roles within the UK primary care workforce^1^ and often the first point of contact for service users.^2^ The patient-facing receptionist role is key to the running of general practice as they help manage demand and facilitate patient access to care.^3–9^ They are often perceived as the ‘gatekeepers’ to primary care services and as a consequence may face hostility from patients.^2,4,7,9–12^ Historically, the receptionist role was centred around answering telephone calls, booking appointments, greeting patients, administering repeat prescriptions, and filing.^5,13^ However, the development of digital technology and the introduction of triage approaches^9,13–16^ has meant the role of the general practice receptionist has evolved over time. Recent UK government plans, which aim to tackle the '8am rush' and make it easier for patients to access primary care services, recognise the developing role of the general practice receptionist as a care navigator and necessitating the development of new skills.^17^

Digital transformation in the NHS has long been promoted by policymakers. In 2018, a UK government policy paper outlining a vision for digital, data, and technology in health and care included promoting online services and for NHS staff to feel empowered and confident in using technology.^18^ However, the COVID-19 pandemic accelerated changes globally, with a rapid increase in the number of people using online services such as patient portals,^19,20^ online prescription ordering,^21^ and remote consultations, particularly video and email.^19,22–27^

Implications of moving to online services have been considered in the literature from a clinician perspective (for example, job satisfaction, administrative burden, and flexibility of working),^19^ but with comparatively little consideration for the impact of changes to the receptionist role in general practice.^28^ One previous ethnographic study found that patients and staff felt that increasing the use of remote consultations would reduce pressure on reception staff; however, there was little evidence to suggest this was the case, and in some respects the workload of reception staff was increased.^3^

This article draws on a team-based focused ethnographic study and interviews with stakeholders to examine the impact that online services have on the role of the general practice receptionist.

## Method

This research was undertaken as part of a wider multi-method study on digital facilitation in primary care (the Di-Facto study) and full details of the methods used are published elsewhere.^29^ For the purposes of the study, digital facilitation was defined as ‘*that range of processes, procedures and personnel which seeks to support NHS patients in their uptake and use of online services’*. Reporting of this study is guided by Standards for Reporting Qualitative Research.^30^

### Qualitative design and context

A team-based focused ethnographic case study and interviews with key stakeholders were conducted between September 2021 and July 2022. The ethnography team consisted of five researchers: a day-to-day lead, a senior lead, and three ethnographers, who worked in the field for up to 6 weeks in each case study practice. The stakeholder interview team consisted of four researchers: a day-to-day lead, senior lead, and two interviewers. Patient and public representatives were involved throughout the Di-Facto study from conception, through the design and conduct of the research, to the synthesis and interpretation of findings.

### Research team

Researchers in the field were trained in observation techniques and were experienced qualitative researchers with varied previous experience of ethnographic approaches. They met fortnightly during data collection and met monthly with experienced researchers. These meetings enabled discussion of the practical aspects of data collection and provided researchers space for reflexivity. Researchers were also encouraged to diarise their reflections with their fieldnotes and share these with team members.

### Sampling and recruitment strategy

This study aimed to include a varied sample of eight practices across a range of primary care settings. Sites were identified through a previous element of the wider study, which included a national practice survey^31^ and via the National Institute for Health and Care Research Clinical Research Networks. Sites were purposefully selected based on their experience and delivery of a variety of digital facilitation approaches and practice characteristics (for example, practice location, Index of Multiple Deprivation score at practice level, list size, and percentage of patients aged ≥65 years). Stakeholders were identified through an initial stakeholder analysis (involving contact mapping using policy review, the research team’s knowledge of the health system and patient and professional bodies, professional networks of the wider research team, and internet searches) and snowball sampling.^32^ The aim was to recruit 12–18 stakeholders with oversight at a local, regional, or national level on matters relating to digital facilitation.

### Data collection methods and procedure

Ethnographic data were collected from eight practices in four regions across England, which involved non-participant observations, document analysis, and interviews with patients or carers and staff. The duration of researchers’ site visits varied depending on the prevalence of the phenomenon of interest (digital facilitation) and continued until no additional data were evident to the researcher across the data sources at each site, and disconfirming views had been obtained.

A case study guide was created (see Supplementary Information S1) to ensure consistency was maintained across the team at multiple sites in data collection. This approach has been used previously in team-based focused ethnography.^33,34^

Extensive handwritten fieldnotes with no identifiers or names were made by researchers and later digitised. Documents (for example, posters, leaflets, and newsletters) relevant to digital facilitation were collected with identifiable information removed, and written descriptions of these were added into the summarised fieldnotes template. As this was a team-based ethnography, it was ensured that data were collated in a standardised format for analysis. This was achieved by using a separate document for each general practice, collating contextual information about each practice, summarised fieldnotes, listing details about who was interviewed, and noting down documentation collected. These documents were updated throughout data collection.

Interviews with patients or carers, staff, and stakeholders were conducted either face to face, via online video conferencing, or telephone, and, with the interviewees’ permission, audio-recorded.

Separate topic guides (see Supplementary Information S2) were developed for staff, patient or carer, and stakeholder interviews. These were informed by a review of the literature^35^ and the survey of general practices.^29^ The staff topic guide covered drivers for supporting online access in the practice, the type of support in use, the perceived success of this assistance, and challenges to implementation. The patient or carer topic guide explored use of online services outside of health, challenges to using online GP services, and participants' experiences of digital support within the practice. The stakeholder topic guide explored key drivers of digital facilitation, perceptions of digital facilitation in practice, concerns around digital inclusion, and the evolving policy context.

### Data processing and analysis

All interviews were audio-recorded, professionally transcribed, and checked for accuracy by the researcher who conducted the interview. Data from the focused ethnography and stakeholder interviews were analysed together using reflexive thematic analysis^36^ through the following steps: 1) reading transcripts and developing coding frames; 2) agreeing a final coding frame at an analysis meeting that worked for all data source types; 3) gathering related sections of transcripts, fieldnotes, and documents under thematic codes; 4) applying thematic analysis to each line of argument in the text, using the ‘one sheet of paper’ method^37^ to create summaries of each code, and grouping codes into broader themes or axial codes from which themes were derived and summarised; and 5) sharing findings with the wider research team to finalise interpretation.

## Results

### Practice context

Practices varied in terms of their location, patient list size, proportion of ethnic minority patients, patients aged ≥65 years, and Index of Multiple Deprivation score (Table 1). Researchers spent 45–76 hours in each practice over a period of 2–6 weeks. They conducted interviews with 33 patients or carers (length: 14–50 minutes) (characteristics in Table 2) and 36 staff (length: 8–60 minutes) between September 2021 and July 2022 (Table 3), and 19 stakeholders (length: 22–62 minutes) between October 2021 and May 2022 (Table 4).

**Table 1.** Characteristics of included practices

Practice ID	List size^a^	Location	Ethnic minority patients, %^b^	Patients aged ≥65 years, %	Index of Multiple Deprivation score	Receptionist title in the practice
A	Large	Semi-rural	4.2	23.4	10 (Low)	Receptionist
B	Small	Urban	85.7	7.3	1 (High)	Receptionist
C	Medium	Urban	40.0	9.4	3 (High)	Receptionist
D	Large	Urban	1.5	23.9	9 (Low)	Receptionist
E	Large	Rural	1.8	8.3	5 (Medium)	Care adviser
F	Large	Rural	1.2	33.4	6 (Medium)	Patient care adviser
G	Large	Urban	6.4	19.6	8 (Low)	Receptionist
H	Small	Urban	1.0	14.7	2 (High)	Patient care adviser

^a^Small = <6000 patients; medium = 6000–12 000 patients; and large = >12 000 patients. ^b^Including Asian/Asian British; Black/African/Caribbean/Black British; Mixed; White; and Other ethnic group, as reported on https://fingertips.phe.org.uk.

**Table 2.** Characteristics of interviewed patients or carers (*N* = 33)

Characteristics	Category	Participants, *n*
Gender	Female	18
	Male	15
Age group, years	18–24	2
	25–34	3
	35–44	4
	45–54	5
	55–64	6
	65–74	6
	75–84	4
	≥85	2
	Undisclosed	1
Ethnicity	White British	26
	Asian	6
	Black Caribbean	1
Health	Long-term condition(s)	13
	Disability	1
Carer	Yes	7
	No	26

**Table 3.** Characteristics of interviewed practice staff (*N* = 36)

Characteristics	Category	Participants, *n*
Gender	Female	23
	Male	13
Age group, years	18–24	3
	25–34	10
	35–44	7
	45–54	7
	55–64	4
	≥65	1
	Unknown	4
Role in practice	Practice manager	8
	Receptionist	6
	Reception manager	1
	Administrator	2
	Data/IT/QC/Business manager	4
	GP	8
	Nurse or healthcare assistant	3
	Paramedic	1
	Clinical pharmacist	2
	Social prescriber	1

IT = information technology. QC = quality control.

**Table 4.** Characteristics of interviewed stakeholders (*N* = 19)

Organisation type	*n*	Level	*n*	Clinical or non-clinical	*n*
NHS infrastructure	9	National	11	Clinical role	10
Third-sector organisation	7	Regional Local	5 3	Non-clinical role	9
Academia	2				
Provider of digital platform	1				

**Figure 1.**
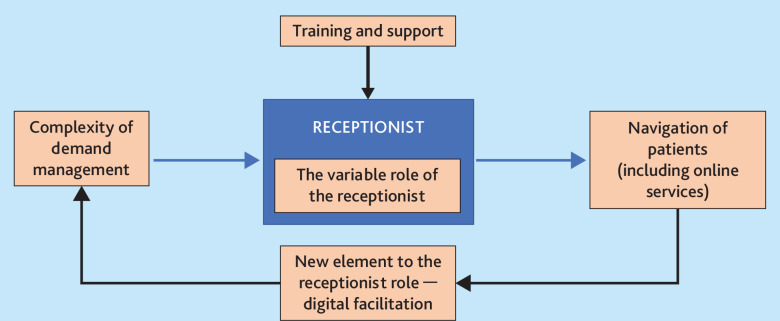
Themes identified highlighted the varied and integrated role of the receptionist.

We identified five themes that highlight the varied, integrated role of the receptionist. The interconnection of these themes are illustrated in Figure 1. The complexity of demand management that online services create directly feeds into the variable role of the receptionist, who then must help navigate patients towards the appropriate care. This, in some instances, may require navigating patients to online services, which can create an additional element in the receptionist role of digital facilitation. This is largely done with minimal formal training and support.

### The variable role of the receptionist

The receptionist role is highly variable between practices, and understanding of the role is inconsistent even within individual practices. Tasks undertaken by receptionists depended on whether the practice had specific administrators (for example, prescription clerks and IT officers), the number of receptionists, and the confidence and competence of the receptionists themselves. The volume of different tasks now undertaken by receptionists means the job is perceived to be more varied:

*'It’s changed, whereas it was very repetitive, there is more variety because we are doing lots of different jobs compared to a few of the same jobs.'* (Practice C, receptionist)

Some practices were highly structured, with rotas for which receptionists were responsible for specific tasks in blocks of time that would rotate. Others had greater flexibility in that receptionists would, on the day, allocate tasks for blocks of time.

### Complexity of demand management

The role is *'not just a case of answering a phone'* (Practice F, receptionist) as there are now multiple routes patients can use to access general practice that are monitored by receptionists. Online channels, such as email, online triage tools, practice websites, and specific apps, are used, some of which were easier for reception staff to navigate than others. Many of the online routes were intended to reduce call volumes:

*'... the reality is that people can’t get through on the telephone and there aren’t enough receptionists.'* (Stakeholder 03)

However, reception staff commented that this has not reduced their workload, rather it is the same workload spread through different channels:

*'Same patients will email that used to phone all the time.'* (Practice A, fieldnote)

Some patients were unaware that receptionists also dealt with online requests, and they believed they were bypassing the receptionist:

*'Well, so it* [email] *gets to the horse’s mouth rather than the receptionist.'* (Practice A, patient 02)

However, in some cases patients were understanding that the reception teams were extremely busy managing the online communication streams. Practices also controlled demand by switching off online access platforms in variable fashion:

*'I think if we didn’t turn the eConsults off at 11 we’d have a lot more.'* (Practice E, lead practice nurse)

### Navigation of patients

Acting as a navigator was a key element of the receptionist role in the participating practices. Reception staff promoted online routes to accessing services, especially when there were no more appointments available for those calling the practice. This avoided the patient needing to call back. Some practices reserved appointment slots for those using online services, and so reception staff could navigate patients via online routes to offer them an appointment.

It was observed that reception staff sometimes appeared stressed, and staff acknowledged the role can be stressful, especially around peak times for patient demand, or when appointments have run out for the day, or when patients are frustrated and short tempered:

*'... it was a typical Monday morning, the phones didn’t stop ringing and many patients came in. The receptionists were all quite stressed, including one who I never see stressed!'* (Practice D, fieldnote)

Online services could be helpful in placating patients by providing an alternative route to access services, reducing stress for receptionists:

*'... we used to get a lot of patients that were very cross with us, because they couldn’t get appointments with the doctors they wanted. But now we can say, "Do an e-consult with 'named’ doctor and they’ll get back to you"* […] *So it, sort of, calms them down a bit.'* (Practice E, care adviser)

### New element to the receptionist role — digital facilitation

There was a perception from stakeholders and some general practice staff that enabling patients to self-serve using online services meant that workload relating to patients was consequently reduced for reception staff. Patient queries can now be answered directly by information on the apps and websites, and patients can in many cases access information from their own medical record online.

These benefits to workload were dependent on patients being able to use online services. There were concerns that introducing online services may lead to more queries; for example, when accessing their medical record for the first time. It was evident that the introduction of online services was reshaping the role of the receptionist:

*'So I end up ringing the GP* [receptionist] *and she, I put her on loudspeaker and then she explained what I had to click on and from that day I know how to do it now.'* (Practice H, patient 02)

Responsibility for supporting patients to use online services was regarded by staff, patients, and stakeholders as the role of the receptionist, and they appeared to be expected to absorb it within their role:

*'I don't know whether other GPs are doing it more than, than me. I think, our time is so precious when we're talking to patients … it’s not something that I tend to get involved with. I would tend to refer them to the reception team to go through that side of things.'* (Practice A, GP partner)

This extra role came with complications. Patients may use the online systems incorrectly, which creates extra work for the reception team to reconcile. For example, with online appointment booking, patients were able to select inappropriate appointment slots, which then had to be rescheduled by receptionists, something they would have done anyway and thus not saving them any work:

*'During the pandemic, the practice made the decision to stop the online appointment booking facility and* [it] *has since remained off* […] *This was because patients booked appointments in unsuitable slots (eg, a blood test with a GP instead of a phlebotomist or nurse). The receptionists used to monitor online bookings.'* (Practice F, fieldnote)

In other instances, receptionists perceived an increase in their workload where patients made attempts to work around the system or where they lacked understanding of how to use the systems:

*'... there’s just too many slots, really, for the patients to access us. And it’s not always the correct one. It could be, they may put something in "ask the practice a question", when really, it’s a repeat prescription request* […] *But at the moment we have to look in everywhere else like, "ask the doctor a question". "Ask the nurse a question." "Ask the practice a question." "Ask reception a question"* […] *So, it does, that wastes a lot of our time.'* (Practice G, prescription clerk)

Duplication of contacts by patients reflected a lack of patient confidence in the online systems and this led to duplication of effort to the detriment of patients and staff:

*'... had somebody do an e-consult the other day, who then emailed the surgery to say, "I’ve done an e-consult" and then phoned the surgery, so that’s three lines of doing admin!'* (Practice G, quality and performance manager)

These examples were seen as evidence that there was a need for patients to be supported to understand how to use the online services in the way they were intended to be used, and that some systems were not as user friendly. This role fell to reception staff.

### Training and support

A consequence of this change to the receptionist role was the need for training and support. It was acknowledged by staff and patients that individual receptionists have different skillsets and abilities when it comes to online systems and ways of working. While there were instances where it was possible to match skillsets to tasks among existing staff, extra training and support was also needed to upskill those who needed it so that they were able and confident to fulfil their roles:

*'... you'll have members of staff who are confident to do that. Others not confident, and so you don’t want them doing it. And it’s, again, it’s about how you, we train up and develop those receptionists to act as not just receptionists and booking appointments, but to start filtering the work to various people.'* (Practice G, practice manager)

There was a perception that younger reception staff were more likely to feel more confident using and troubleshooting technology, and therefore may be better placed to support patients:

*'... we’re a bit older now, our workforce downstairs, but we have the youngsters who are really good, you know, you’d say, "Oh, can you sort this out?"'* (Practice E, care adviser)

However, in some circumstances receptionists’ ability to understand the problem and support patients was limited by the online service being provided by a third party:

*'The receptionist said "we don’t have any dealings with the NHS app."'* (Practice H, fieldnote)

In addition, the evolving skillset receptionists now require was thought to be important to consider when hiring new staff, potentially changing recruitment in general practice:

*'... over time we've got to completely change the requirements of general practice reception and admin teams, what we expect from them.'* (Practice G, practice manager*)*

We observed a lack of formal standardised training and support provided to receptionists regarding using online services, with many learning on the job from other receptionists and through trial and error:

*'I wouldn’t say it’s much training, it’s more a sort of, like, we look at it ourselves and find out ourselves* […] *A lot of it’s self-taught.'* (Practice G, receptionist)

In some cases, new systems were brought in and no training was provided. One member of staff indicated that this lack of training, including lack of time and resource, was common:

*'It’s the way with the NHS, you get no training about anything. And so you just accept, you’re expected to pick these things up and use them and know how to advise patients if they can’t use them* […] *we haven’t had any resources to do that. We’ve had no materials, no money really and no facilities to train staff unless we go out and find our own training materials.'* (Practice A, practice manager)

This non-standardised ‘training’ resulted in inconsistent messaging and service provision to patients:

*'... they’d just tell me different things each time I would call up because it would be somebody else at reception.'* (Practice B, patient 05)

New staff reported confusion and concern about how to complete tasks if they had been shown by different people doing it different ways:

*'Apprentice said it is "nerve racking" for her. She has had some training from two different staff but both do it differently.'* (Practice B, fieldnote)

While, generally, receptionists were observed to be good at supporting each other when issues arose or showing new staff the systems, standardised training and policies would be valued.

Stakeholders, on the other hand, believed there was non-mandatory training available but that practices did not always take it up owing to time pressures. In reality there was rarely protected time for receptionists to engage in this training and so it was done when it could be slotted in among other duties:

*'I noticed one of the receptionists had received an email from the* [NAME] *App which was giving notice of the December update* […] *Receptionist 4 said she doesn’t have time to read the update emails about the App.'* (Practice B, fieldnote)

When training did occur, it would sometimes constitute an initial session for a new online service, provided by staff internally or technology providers, meaning that staff missing this or joining afterwards would not receive the training. There would also be mandatory courses relating to important concepts such as information security. Resources for training were sometimes stored on internal drives at the practice, but use of these as training aids was largely not observed or reported during the study. There was one example where guidance was printed and a staff member used it to complete a task, indicating that if it is readily available then it is more likely to be used.

## Discussion

### Summary

The receptionist role within general practice has evolved and become more complex. In our study, the role appears to be different across practices and there appears to be some tailoring of tasks to suit individual skillsets, which happens organically, especially in relation to online services. The role is prone to external influences meaning receptionists lack control over what their role is; for instance, online services are potentially reshaping the receptionist role by changing patient behaviours.

Receptionists are managing multiple modes of communication from patients, and patients lack awareness that online services have been absorbed into this role. The complexity extends to navigation as there are several options available when patients make contact, including referring patients to online services. This has created a new element to the role that involves supporting patients to self-serve via online services. If used by patients as they were intended it reduced workload for the receptionist; however, when used incorrectly it increased workload. The role was regarded as sometimes stressful, particularly when dealing with frustrated patients, but online services provided options to placate patients.

The changing role, particularly in respect of online opportunities, is largely navigated by the receptionists without formal training and staff are mostly expected to learn on the job from other receptionists. There was a general lack of instructional documentation that could be used by staff, and in cases where it did exist, reception staff were largely unaware of it. This led to inconsistencies in practice between staff, which sometimes frustrated patients.

### Strengths and limitations

A strength of this work is the range of data sources used as part of the focused ethnographic case study, which allowed researchers to triangulate information and delve deeper into inconsistencies that utilising a single method would not have achieved. In addition, the eight practices included a variety of geographic locations and socioeconomic demographic characteristics of England as a whole and of the populations they served. Stakeholder interviews provided views beyond individual practices and greater context.

A limitation of this study was that the ethnographic research was conducted during the latter period of the COVID-19 pandemic, which may have impacted patient flow and the receptionist tasks, which may not continue in the same way. In addition, owing to the focus of the work, no data were sought on job satisfaction, retention, or wellbeing of reception staff, which would have provided greater insight into the impact of online services on this role. However, a recent ethnographic study reported receptionists ‘performing’ their role as if on stage, hiding their true identities behind customer service and inflexible digital policies, with many leaving their job because digitalisation had led to increased workload and hostile working conditions;^12^ and this study did not consider the additional task of digital facilitation.

### Comparison with existing literature

The current state of general practice provided important wider context for the findings of this study. Staff in the study had noticed a growth in patient numbers and perceived a corresponding increase in their workload. Consultation rates increased by 15% since 2018–2019 to when the data in the present study were collected in 2021–2022.^38^ The impact of the COVID-19 pandemic was still felt at the time of the fieldwork with increased workload relating to vaccinations and COVID passports, and some practices experienced staff shortages. Increasing demands for general practice services without capacity to meet this demand is a national issue for the UK.^39^ Central to the NHS England plan to manage demand by tackling the ‘8am rush’, and within the 2023–2024 GP contract, is to avoid telling patients to call back at a later time;^17,40^ however, we observed this already happening in practices, with many receptionists recommending patients access via online services instead of calling back.

The receptionist role is complex, demanding, and stressful,^11,41^ and involves high levels of task and skill variety as well as information processing.^42^ The present study suggests that the evolution of online services may actually amplify this complexity. There are few studies reporting the effects online services have specifically on the general practice receptionist role and this article contributes to the literature by addressing this gap and explicitly acknowledging the task of digital facilitation within the receptionist role.

Previous work on alternatives to face-to-face consultations in general practice suggested that the introduction of any new technology can be highly disruptive to practices, in terms of organisational, professional, and spatial disruptions and dynamics, and can also have unintended consequences.^28^ From this study, for instance, patients making multiple contacts and inappropriately using online services required extra administration and coordination between staff. Similarly, a recent ethnographic study found that the digitalisation of UK general practice facilitates increased access, which for some patients can lead to excessive use and a supply-induced demand,^12^ which receptionists often have to manage. However, there were also cases in the present study where technology streamlined tasks; for instance, the ability to send mass text messages or links to online services, also found in the ethnographic study.^12^ This reflects that digital approaches are more likely adopted when they serve a purpose and meet a need.^43,44^

Previous research suggested that the receptionist role began changing before the COVID-19 pandemic with services moving towards a more remote online model, which required some receptionist teams to be re-trained, resulting in some leaving because they no longer enjoyed the role.^3^

In the 1980s many general practice receptionists did not undergo formal training and the majority learnt on the job from other reception staff, practice managers, or sometimes clinical staff,^4,5^ and for some aspects and/or individuals in the role this largely remains the case 40 years later.^3,9,11,13,42^

The rapidity of changes required owing to the COVID-19 pandemic, especially related to online services, may have resulted in training being bypassed or fast-tracked,^19^ which may be a contributing factor in the present study. There is a need to recognise, formalise, and support the receptionist role in remote triage and care navigation,^13,17,45^ which considers the multiple channels by which patients can now enter general practice. A lack of time and funding were commonly mentioned barriers to training for receptionists in the present study and previous research.^13,45^

### Implications for research and practice

The evolving role of the general practice receptionist requires skills and capabilities to competently use and monitor multiple online services to manage patient demands and navigate patients towards appropriate services. While the COVID-19 pandemic expedited the use of online services for some general practices, many continue to use them post-pandemic, indicating a lasting change. This may have implications for recruitment strategies for practices (for example, job descriptions and skills requirements). Updated guidance on the role, expectations, and skills requirements of receptionists may be helpful, especially as the role has potential to become more professionalised. For those newly recruited or already in post, more formal support and training for reception staff may be required, if even to standardise messaging and practices within a general practice, which requires further research. Future research is needed to investigate the retention, job satisfaction, workload, and recruitment processes, which may be appropriate in respect of the evolving role of receptionists. In addition, research looking at system-level changes should consider the impacts of the changes on this role.

## Data

Study data may be made available to appropriate individuals on a case-by-case basis following an application to the Chief Investigator (via the corresponding author).
